# Occult constrictive pericardial disease emerging 40 years after chest radiation therapy: a case report

**DOI:** 10.1186/s12872-018-0848-7

**Published:** 2018-05-31

**Authors:** Chiaki Goten, Hisayoshi Murai, Shin-ichiro Takashima, Takeshi Kato, Soichiro Usui, Hiroshi Furusho, Takahiro Saeki, Satoru Sakagami, Hirofumi Takemura, Shuichi Kaneko, Masayuki Takamura

**Affiliations:** 1 Department of System Biology, Graduate School of Medical Science, Kanazawa University, 13-1 Takara-machi, Kanazawa, Ishikawa 920-8641 Japan; 2 Department of Cardiology, National Hospital Organization, Kanazawa Medical Center, 1-1 Shimoishibiki-machi, Kanazawa, Ishikawa 920-8650 Japan; 3 Department of Thoracic, Cardiovascular and General Surgery, Kanazawa University, 13-1 Takara-machi, Kanazawa, Ishikawa 920-8641 Japan

**Keywords:** Occult constrictive pericardial disease, Valvular disease, Radiation therapy

## Abstract

**Background:**

The main etiology of constrictive pericarditis (CP) has changed from tuberculosis to therapeutic mediastinal radiation and cardiac surgery. Occult constrictive pericardial disease (OCPD) is a covert disease in which CP is manifested in a condition of volume overload.

**Case presentation:**

A 60-year-old patient with a history of thoracic radiation therapy for non-Hodgkin’s lymphoma (40 years earlier) was transferred to our hospital for treatment of repeated congestive heart failure. For a preoperative hemodynamic study, pre-hydration with intravenous normal saline (50 mL/hour) was used to manifest the pericardial disease and prevent contrast-induced nephropathy. The hemodynamic study showed a right ventricular dip-plateau pattern and discordance of right and left ventricular systolic pressures during inspiration, which was not seen in the volume-controlled state. These responses were concordant with OCPD. A pericardiectomy, aortic valve replacement, and mitral and tricuspid valve repair were performed. Postoperatively, the heart failure was controlled with standard medication.

**Conclusions:**

This case revealed a volume-induced change in hemodynamics in OCPD with severe combined valvular heart disease, which suggests the importance of considering OCPD in patients who had undergone radiation therapy 40 years before.

## Background

Occult constrictive pericardial disease (OCPD) is a rare disease characterized by a change in hemodynamics to a typical constrictive pericarditis (CP) pattern after volume loading [1]. The main causes of CP are idiopathic (46%), previous thoracic surgery (37%), and radiation therapy (9%) [2, 3]. The incidence of complications after thoracic radiation therapy for tumors has increased with the use of therapy for thoracic tumors. About 7–20% of patients develop chronic pericarditis 10 or more years after radiation treatment [4]. The average onset time of valvular disease with or without symptoms after thoracic radiation therapy is 11.5 and 16.5 years, respectively [5]. Radiation therapy induces microcirculation injury with endothelial damage, neovascularization, and atherosclerosis, which lead to fibrosis and calcification of valves and pericardium. Here, we report a patient with heart failure and OCPD complicated by severe valvular disease 40 years after thoracic radiation therapy for non-Hodgkin’s lymphoma.

## Case presentation

A 60-year-old woman presented to another hospital with shortness of breath on exertion. Her family history was unremarkable. She had undergone chemo-radiation for non-Hodgkin’s lymphoma 40 years earlier and achieved complete remission. CHOP (cyclophosphamide, doxorubicin, vincristine, and prednisone) chemotherapy did not induce cardiac toxicity. Twenty years earlier, she had an anterior myocardial infarction and underwent percutaneous coronary intervention. Moderate combined valvular diseases (aortic regurgitation, mitral regurgitation, and tricuspid insufficiency) were also followed. She had received standard treatment for chronic heart failure. However, she had suffered from acute exacerbation of congestive heart failure several times beginning 2 years before and was referred to our hospital because of refractory repeated congestive heart failure.

Her vital signs were blood pressure 106/43 mmHg, heart rate 72/min, and SPO_2_ 97% on room air, with a body mass index of 15.8 kg/m^2^. The conjunctiva showed no signs of anemia or jaundice. The first and second heart sounds were normal, but the third one was increased. There was a pan-systolic murmur at the cardiac apex (Levine III/VI). There were no rales in the lungs. The abdomen was flat and soft and the liver was palpated two finger widths below the ribs. There was no edema, ascites or coldness of the limbs. She had hepatomegaly and jugular vein distention remarkable at inspiration, also known as “Kussmaul’s sign”.

Laboratory tests revealed mild renal and liver dysfunction, and the brain natriuretic peptide (BNP) level was 492 pg/mL. There were no signs of an inflammatory response, anemia, or thyroid dysfunction. Chest X-ray showed a cardiothoracic ratio of 47% and no evidence of pleural effusion, pulmonary congestion, or pericardial calcification. The electrocardiogram showed a pulmonary P wave, high left-side voltages, poor R progression, and ST changes in V_5_ and V_6_.

**Fig. 1:**
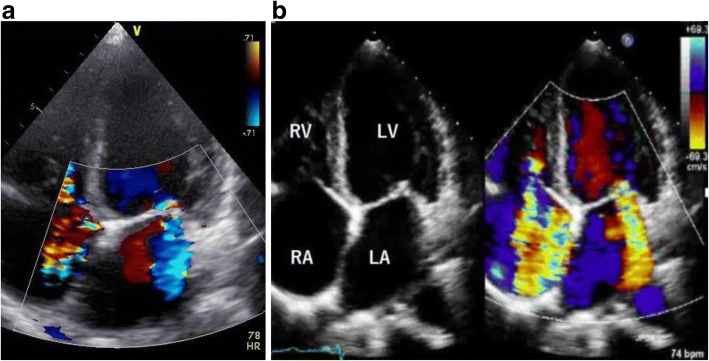
Color-Doppler Echocardiography of the mitral and tricuspid valves. There was a worsening of both mitral and tricuspid regurgitation in the last hospital admission (**b**) compared to at the end of previous hospitalization (**a**). RA, right atrium; RV, right ventricle; LA, left atrium; LV, left ventricle

Echocardiography showed decreased motion of the left anterior septum and the left ventricular ejection fraction (LVEF) was 42%. There was bilateral atrial enlargement, but no left ventricular dilatation. No pericardial effusion was seen. Although moderate mitral regurgitation, and tricuspid insufficiency were seen at the end of a previous hospitalization (Fig. 1a), severe mitral and tricuspid regurgitation were detected at our hospital admission (Fig. 1b). The septal leaflet of the tricuspid valve showed decreased mobility. The tricuspid regurgitation pressure gradient was 45 mmHg. Systolic flow reversal in the hepatic veins was observed, suggesting severe tricuspid regurgitation. Pulse doppler flow showed that the ratio of mitral peak velocity of early filling (E: 1.45 m/s) to late filling (A: 0.98 m/s) was 1.5, and the deceleration time was 211 ms. The ratio of mitral peak velocity of early filling (E) to early diastolic mitral annular velocity (E’: 6.9 cm/s) was 21. The early diastolic left ventricular filling velocity was reduced by 27% during inspiration. These findings indicate the possibility of CP and/or restrictive cardiomyopathy. The inferior vena cava was enlarged to 22 mm and the respiratory fluctuation was decreased. We diagnosed acutely developed congestive heart failure due to severe mitral and tricuspid regurgitation, which was not observed at the end of hospitalization at the previous hospital. The patient was prescribed additional diuretics to reduce volume overload.

**Fig. 2:**
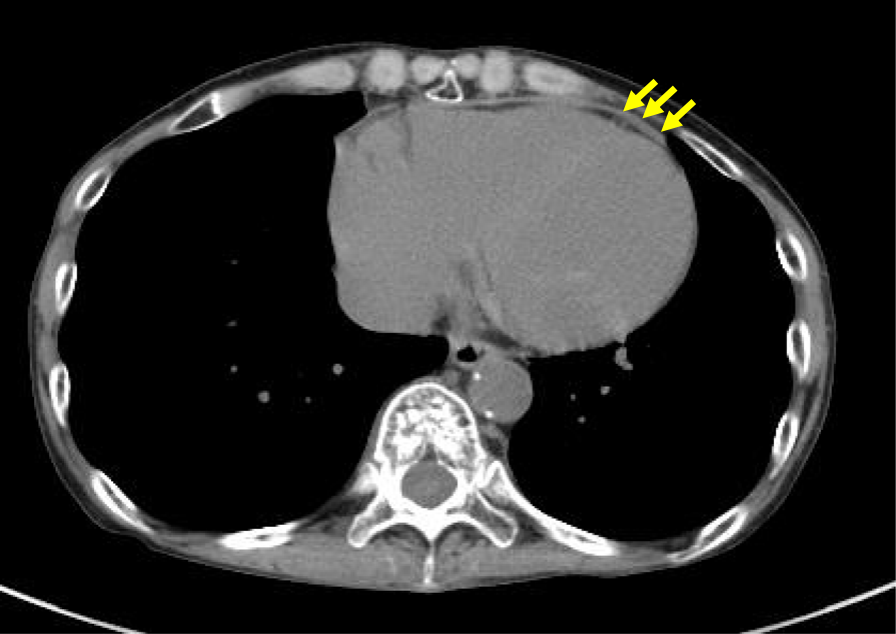
Computed tomography showed moderate pericardial thickening. (yellow arrow)

Computed tomography revealed a mild pericardial thickening of 4 mm in front of the right ventricle (Fig. 2). Considering the history of radiation therapy, it was likely that the patient was suffering from concealed pericardial disease.

**Fig. 3:**
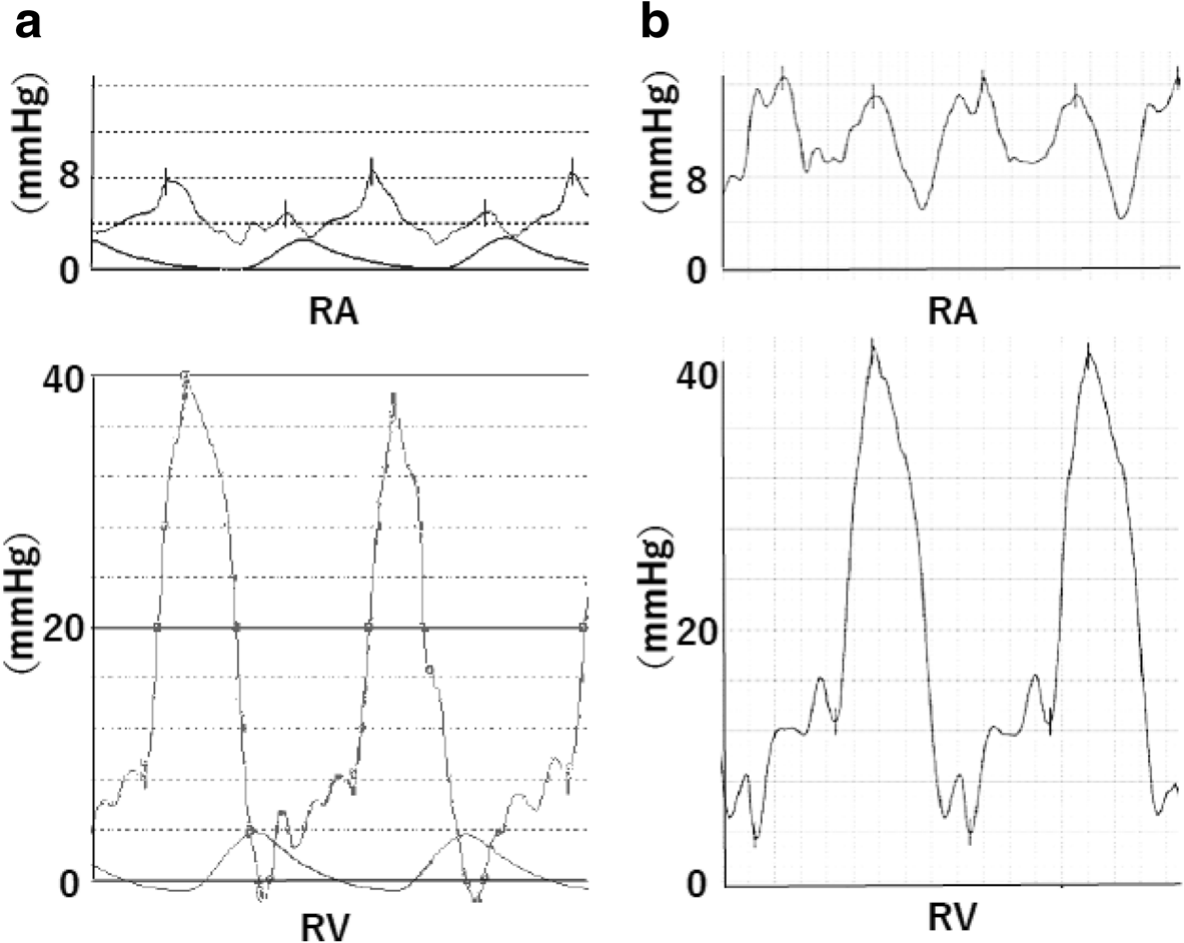
Right heart catheterization showed a normal pressure pattern in a previous pressure study (**a**). In the current study, the right ventricular systolic pressure exceeded 40 mmHg and there was a dip and plateau pattern (**b**). RA, right atrium; RV, right ventricle

**Fig. 4:**
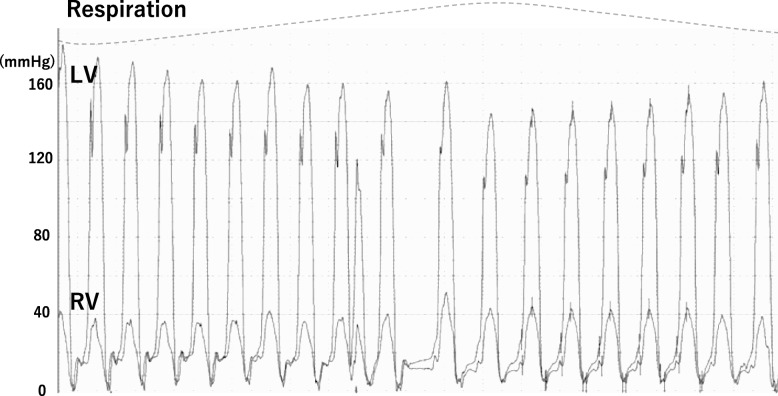
The systolic pressure was dissociated between the two ventricles and showed mirror-image discordance consistent with constrictive pericarditis. RV, right ventricle; LV, left ventricle

Prior to the cardiac catheter examination, normal saline was infused at a rate of 50 mL/h for 12 h to manifest the pericardial disease and to protect renal function from the contrast medium. Coronary angiography showed that the coronary arteries were intact and there was a stent in the left anterior descending artery. The right atrial, end diastolic right ventricular, diastolic pulmonary artery, and pulmonary capillary wedge pressures were 15 mmHg. This finding was not seen in a right heart pressure study performed previously in hospital about 6 months ago (Fig. 3a). In the right atrial pressure waveform there were prominent x and y descents. The right ventricular systolic pressure exceeded 40 mmHg and showed a dip-plateau pattern (Fig. 3b). In the simultaneous pressure measurement of the left and right ventricle, the difference in end-diastolic pressure was within 5 mmHg. At maximum inspiration, the systolic pressure of both ventricles was dissociated, and showed mirror-image discordance (Fig. 4). Based on these changes in hemodynamics after loading of the patient with fluids, we revealed the diagnosis of OCPD.

Pericardiectomy, aortic valve replacement, and mitral and tricuspid valvuloplasty were performed. At surgery, there was a hard, thickened pericardium, and very mild pericardial adhesion. Histological examination of the resected pericardium revealed hyaline-like fibrous hypertrophy.

Twelve months postoperatively, there was no acute exacerbation of the congestive heart failure. The New York Heart Association functional class had also improved from III to II. On echocardiography, although mild tricuspid and mitral regurgitation remained, the size of both the left and right atria decreased. The percent difference in E-velocity between expiration and inspiration was improved to 12% after pericardiectomy, compared to 27% before.

## Discussion and conclusions

To the best of our knowledge, this is the first report of OCPD emerging in a patient who had undergone radiation therapy 40 years ago. The patient was considered to be suffering from radiation-induced pericardial disease as well as valvular heart disease. Veinot et al. reported that the frequency of pericardial disease was as high as 70% when thoracic surgery was performed in patients with radiotherapy [6]. This implies that some cases of CP might not be diagnosed in routine practice. Bush et al. reported that intravenous drips were useful for detecting OCPD [1]. They rapidly injected 1000 mL of warmed normal saline intravenously over 6 to 8 min and performed right heart catheterization. In our case, we applied non-rapid volume overload because of the adverse effects on heart failure. However, a constrictive physiology pattern was manifested, even using this slow volume injection. Our findings suggest that the volume of injection should be different among patients with congestive heart failure who suspected OCPD.

A previous report demonstrated that valvular disease is seen in about 15% of patients within 40 years after radiation therapy for Hodgkin’s lymphoma. Valvular disease is more frequent with radiation doses ≥30 Gy, and the frequency of valvular disease increases with time and radiation dose [7]. Although the underlying mechanisms are unclear, aortic and mitral valve diseases are common, while tricuspid and pulmonary valve disease are infrequent.

Right ventricular pressure waveform is important to determine the accurate diagnosis for heart failure. However, in some cases of heart failure, the right ventricular waveform often shows a dip-plateau pattern on the right heart catheterization. Both right and left ventricular pressures should be measured simultaneously to distinguish between heart failure and CP. Hurrell et al. demonstrated that the right and left ventricular pressures in heart failure decrease concordantly during inspiration, while the left ventricular pressure decreases and the right ventricular pressure increases during inspiration in CP [8]. Therefore, simultaneous both ventricular pressure measurement is needed in patients with refractory heart failure with a history of chest surgery or radiotherapy.

In decompensated heart failure, chronic mitral regurgitation generally deteriorates in response to prominent left ventricular enlargement [9]. In our case, increased mitral regurgitation was not accompanied by an increase in left ventricular end diastolic diameter. It may be also important point to suspect OCPD if left ventricular dilatation is not observed in a patient with worsening valvular disease.

Although there are no recommended therapies to date for OCPD, the treatment generally follows the guidelines for CP [10]. In our case, a pericardiectomy was done because it was necessary to perform surgery for severe combined valvular heart disease. The European Society for Cardiology guidelines state that the mortality rate of pericardiectomy for CP is as high as 6–12% per year. The major complications of pericardiectomy are acute heart failure and heart rupture [10]. Busch et al. reported that reduced LVEF and right ventricular dilatation were independent predictors of early mortality, whereas coronary artery disease, chronic obstructive pulmonary disease and renal insufficiency were risk factors for late mortality [11]. Our case showed reduced LVEF (42%) and myocardial infarction, which may be related to the mortality. Our patient had a satisfactory clinical course with no heart failure at 12 months postoperatively.

We experienced a rare case of OCPD with severe combined valvular heart disease 40 years after thoracic radiation therapy for non-Hodgkin’s lymphoma. In this case, OCPD emerged due to volume overload. Therefore, we should consider OCPD in patients who have undergone mediastinal radiation therapy, even decades later.

## Abbreviations

CP: Constrictive pericarditis
LA: Left atrium
LV: Left ventricle
OCPD: Occult constrictive pericardial disease
PISA: Proximal isovelocity surface area
RA: Right atrium
RV: Right ventricle
TRPG: Tricuspid regurgitation pressure gradient
